# Supplementary material for: Nonlinear dynamics and chaos in an optomechanical beam
Source: Nat Commun. 2017 Apr 11;8:14965. doi: 10.1038/ncomms14965 (PMC5394270; doi:10.1038/ncomms14965)
Supplement: Supplementary Information — Supplementary Figures, Supplementary Discussion and Supplementary References [file ncomms14965-s1.pdf]

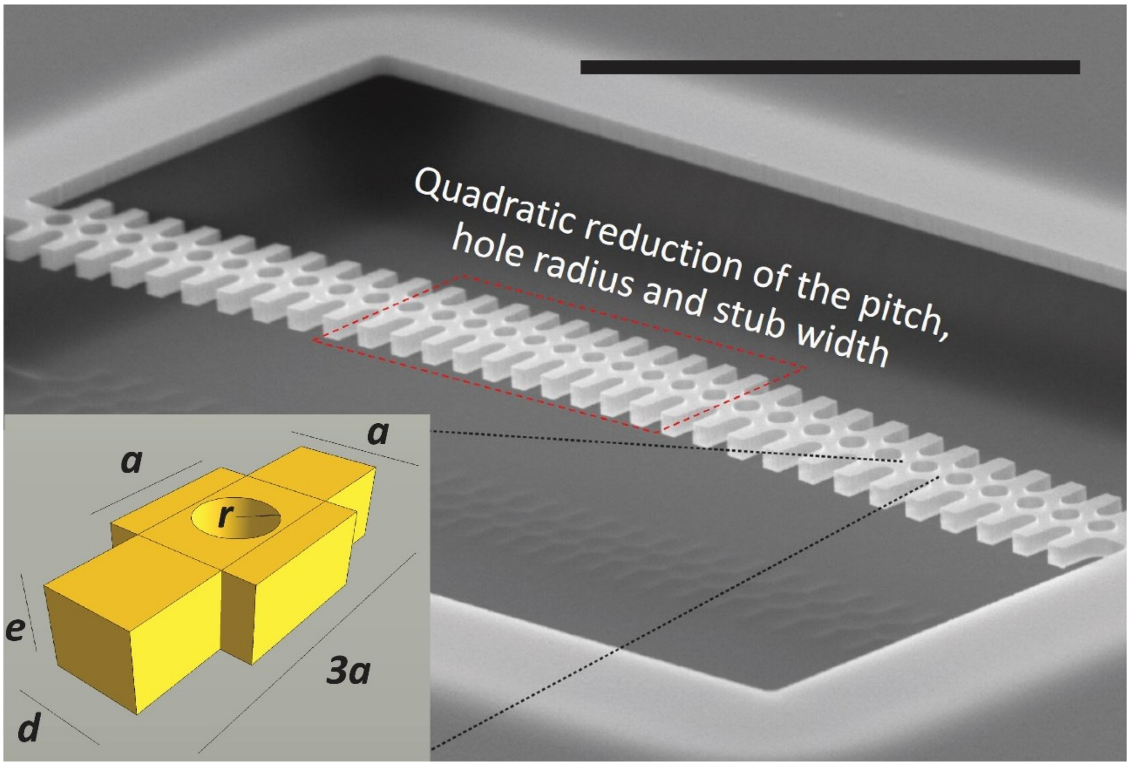

Supplementary Figure 1. An OM photonic crystal. The unit-cell contains a hole in the middle and two symmetric stubs on the sides (inset). Its specific geometry is defined by the size of the square side (*a*), the radius of the hole (*r*), the stubs length (*d*), the cell thickness (*e*) and the total width of the cell (*3a*). The nominal geometrical values of the cells of the mirror are *a*=500 nm, *r*=150 nm, *d*=250 nm and e=220 nm. The defect region consists of 12 central cells, in which the pitch (*a*), *r* and *d* are decreased in a quadratic way towards the centre. The maximum reduction of those parameters is denoted by Γ. A 10 period mirror is included at both sides of the defect region. The main panel illustrates a geometry with Γ=83%. The scale bar represents 5 μm.

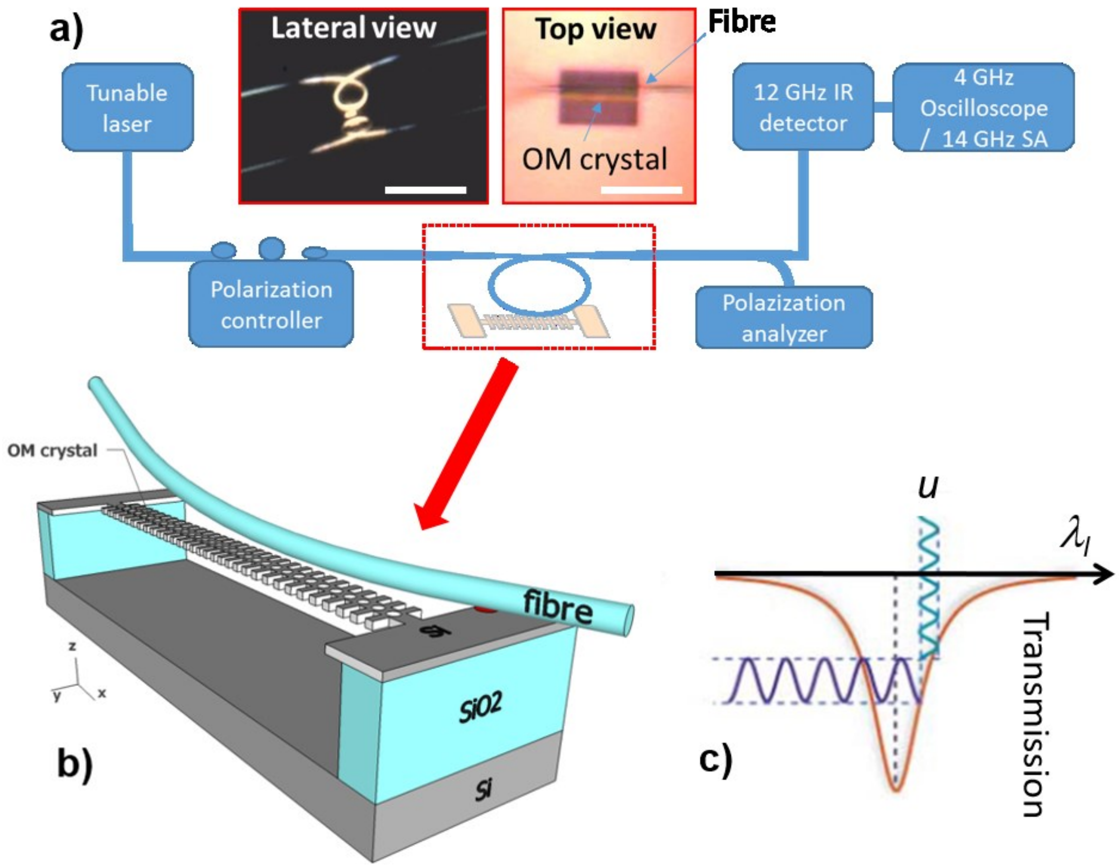

Supplementary Figure 2. Experimental setup and transduction principle. a). Sketch of the experimental setup to measure the optical and mechanical properties of the OM devices. The sample size has been greatly increased for clarity. The top left photo shows a lateral view of the real microlooped tapered fibre close to the sample (scale bar ~50 μm), where the fibre can be seen reflected on the sample. The top right photo shows a top view of the tapered fibre placed parallel to the OM structure and in contact with one of the edges of the etched frame (scale bar ~15 μm). b). Relative positioning of the tapered fibre and the OM photonic crystal. The leaning point of the fibre is highlighted in red. The fibre is placed close enough to the central part of the OM photonic crystal to excite efficiently its localized photonic modes. c) Scheme of the transduction principle. Mechanical fluctuations affect the resonance position (light blue oscillating signal), which lead to intensity fluctuations on the transmitted signal (dark blue oscillating signal).

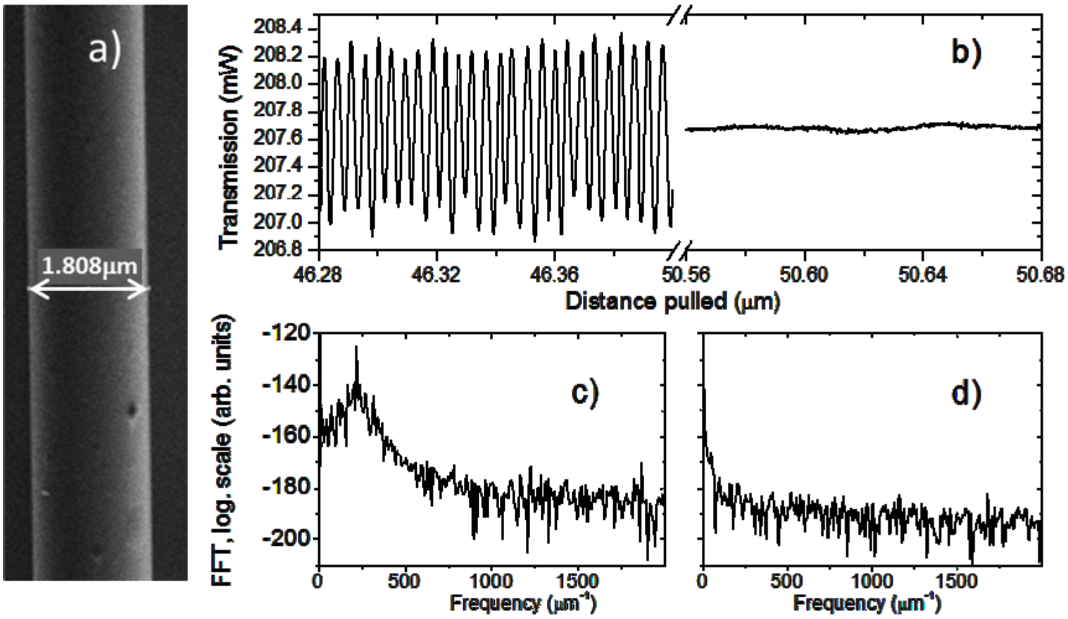

Supplementary Figure 3. Tapered fibre fabrication. a) SEM image of the thinnest part of a tapered fibre. b) Transition from multimode to monomode while pulling the two extremes of the fibre as a function of the pulled distance. c) Fast Fourier Transform of the transmitted signal before the transition to single mode. d) Fast Fourier Transform of the transmitted signal after the transition to single mode.

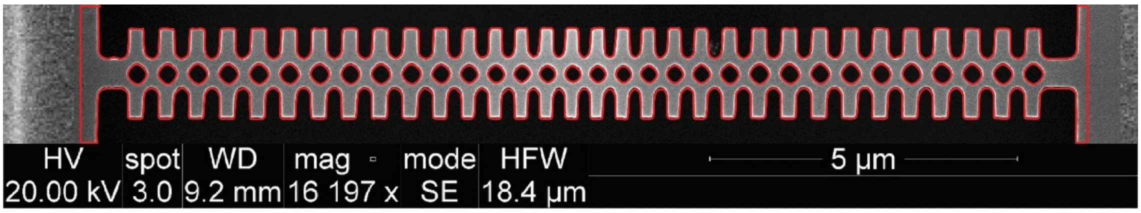

Supplementary Figure 4. Geometrical contour imported by the FEM solver. Top-view SEM micrograph of the OM system for extracting geometrical contour (in red) imported by the FEM solver.

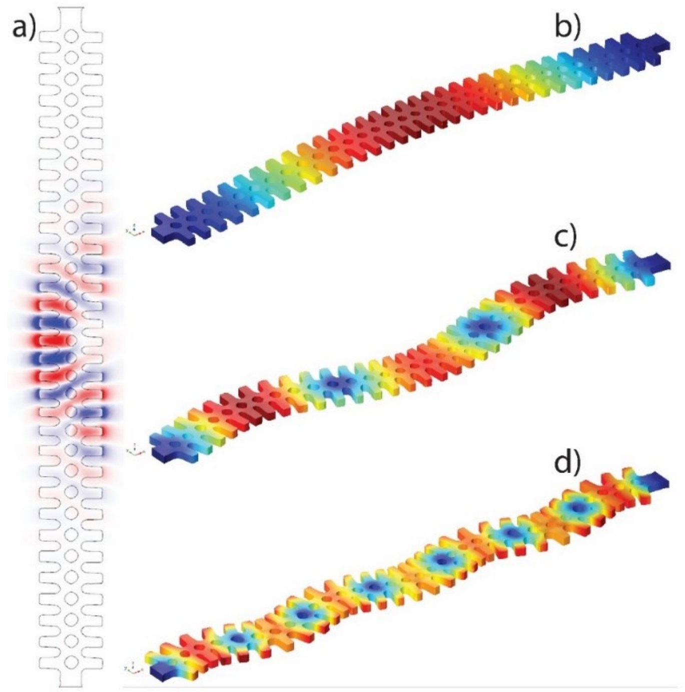

Supplementary Figure 5. Relevant optical and mechanical modes. Panel a) Normalized optical $\mathbf{E}_y$ field of the third optical mode supported by the OM crystal. Panel b), c) and d). Normalized mechanical displacement field $|\mathbf{Q}|$ of the Ω_m'=5 MHz, Ω_m=54 MHz and 198 MHz mechanical modes.

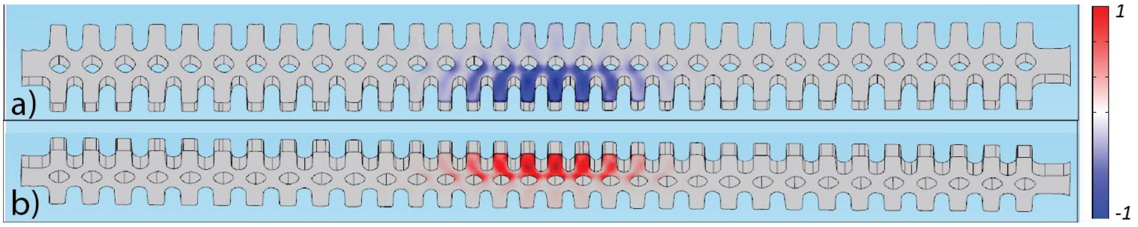

Supplementary Figure 6. Moving boundary contribution for the mechanical mode at Ω_m'=5 MHz. Normalized surface density of the integrand in Equation 2 for the Ω_m'=5 MHz mechanical mode, showing the contributions to g_{MI} of the top and bottom air-Silicon interfaces (panels a) and b), respectively). Both contributions are equivalent in magnitude but opposite in sign, thus leading to an overall $g_{MI}/2\pi$ value of only 7 kHz.

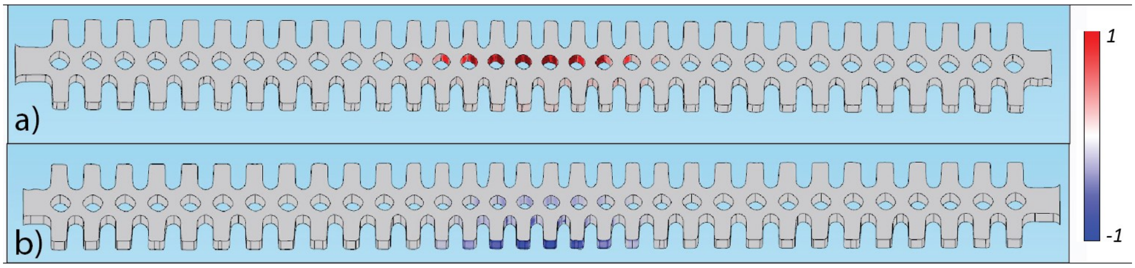

Supplementary Figure 7. Moving boundary contribution for the mechanical mode at Ω_m=54 MHz. Normalized surface density of the integrand in Equation 2 for the Ω_m=54 MHz mechanical mode, showing the contributions to g_{MI} while observing the structure from the top and from the bottom (panels a) and b) respectively). Although the contribution from the wings surface and the hole surface have opposite signs, the former is much larger in magnitude, thus giving rise to an overall $g_{MI}/2\pi$ of 650 kHz.

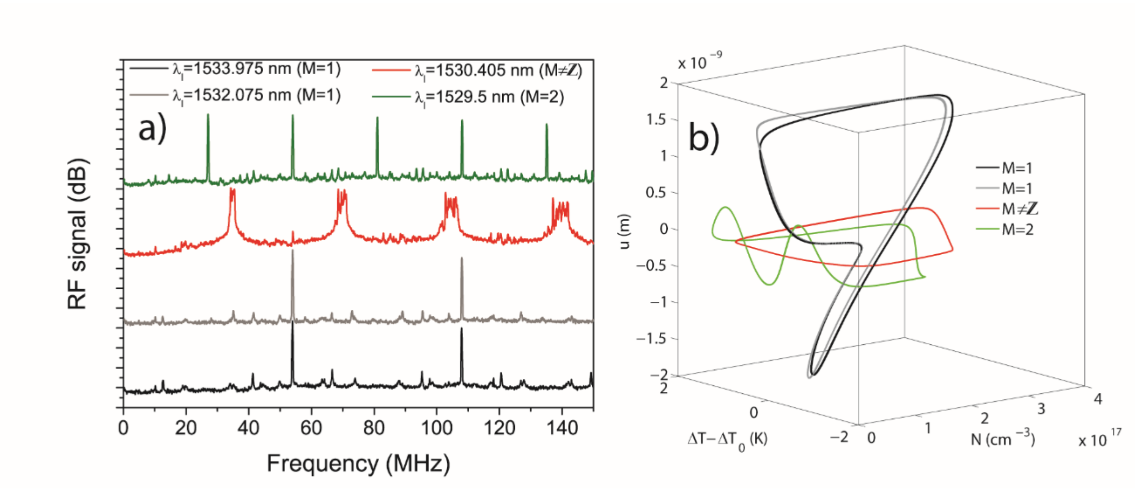

Supplementary Figure 8. Phonon lasing and self-pulsing. a) Experimental RF spectrum at different values of λ_l. The black and grey curves correspond to experimental RF spectra of a SP/phonon lasing regime involving a mechanical mode at Ω_m=54 MHz in the two extremes of the M=1 plateau. The green curve corresponds to SP/phonon lasing with M=2, in which the mechanical oscillation at frequency Ω_m is superimposed on a SP trace at frequency Ω_m/2. Although the black and grey curves are obtained at two different values of λ_l, the signal is locked at the same frequency Ω_m. Here it becomes evident that the mechanical oscillator is not only pumped resonantly, but also that the large amplitude of the coherent mechanical motion acts as a feedback that stabilizes and entrains the SP and the mechanical oscillator. Since Ω_m is much more robust than ν_{SP}, the SP mechanism adapts its frequency to the mechanical one. When the resonant condition with the mechanical oscillation is not fulfilled (red curve) the RF peaks in a frequency-unlocked region are inhomogenously broadened in frequency because the integration time is much greater than the typical period of the signal. b) Simulated phase portraits calculated using the Equations 5, 6 and 7 for equivalent situations to those of panel a). Coherent mechanical oscillations of nanometric amplitude are obtained in the M=1 and M=2 cases, while the red curve is flat in the u-axis.

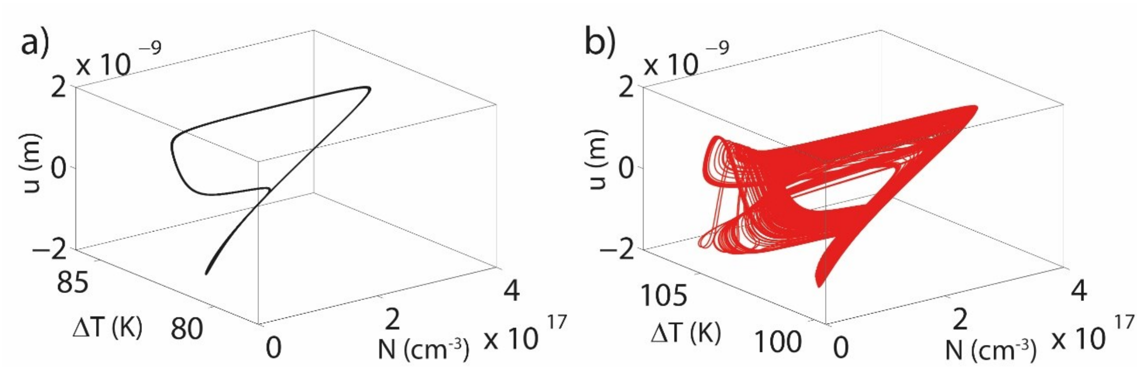

Supplementary Figure 9. Numerical simulations of phase-space trajectories of SP/phonon lasing and chaos. a) Simulated limit cycle associated with a four-dimensional SP/phonon lasing (λ_l=1531.5 nm). b) Simulated trajectory associated with a chaotic regime (λ_l=1532.7 nm). The trajectory tends to fill a restricted volume of the phase space. The maximum intracavity photon number in both cases is n_o=10^5.

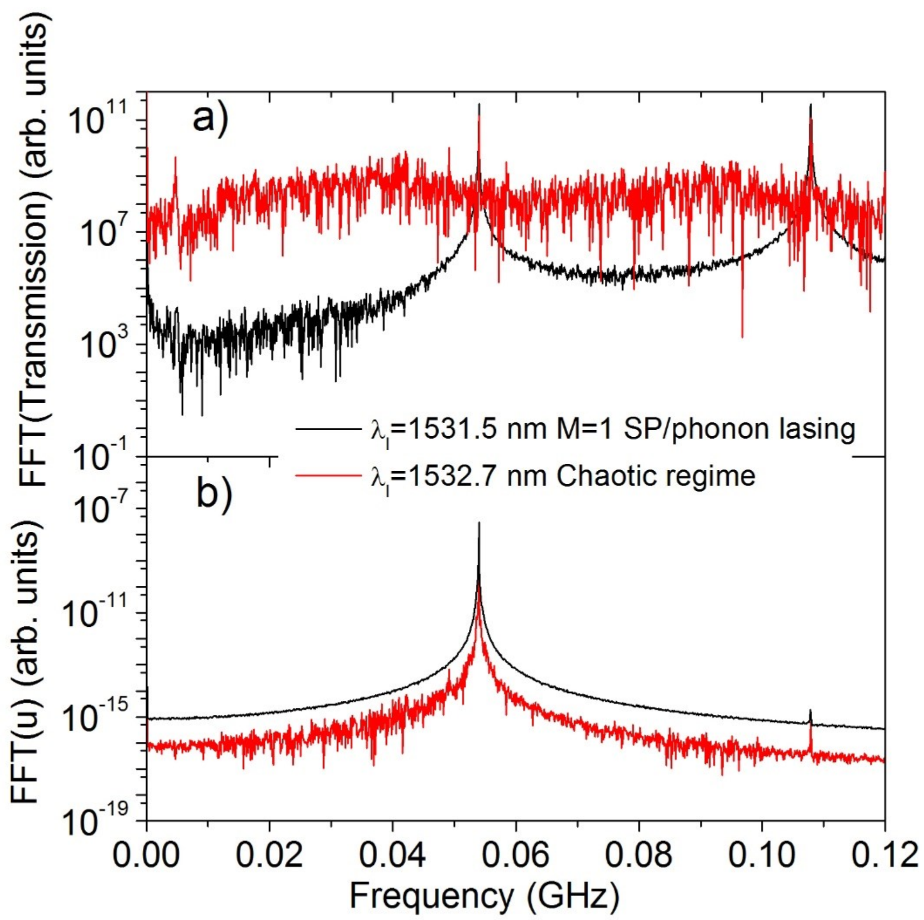

Supplementary Figure 10. Simulated frequency spectra in the SP/phonon lasing regime and in the chaotic regime considering two mechanical oscillators. Fast Fourier Transform of the simulated transmitted signal (panel a)) and of the deformation of the first oscillator (*u*, panel b)), which is the one with Ω_m=54 MHz. The black curves are associated with the four-dimensional SP/phonon lasing regime (λ_l=1531.5 nm) while the red curved are associated with the chaotic regime (λ_l=1532.7 nm). In the latter case, it is worth to note that the spectrum of the transmitted signal is broad band, as expected from a chaotic regime. On top of it there are peaks associated with the two mechanical modes in consideration (Ω_m=54 MHz and Ω_m'=5 MHz), thus indicating that both of them are in a regime of high amplitude oscillations. Interestingly, the Fast Fourier Transform of the deformation of the first oscillator (red curve of panel b)) indicates that the mechanical oscillation is coherent. On the contrary, the Fast Fourier Transform of either ΔT or N is broad band, that is, chaotic. The maximum intracavity photon number in all cases is n_o=10^5.

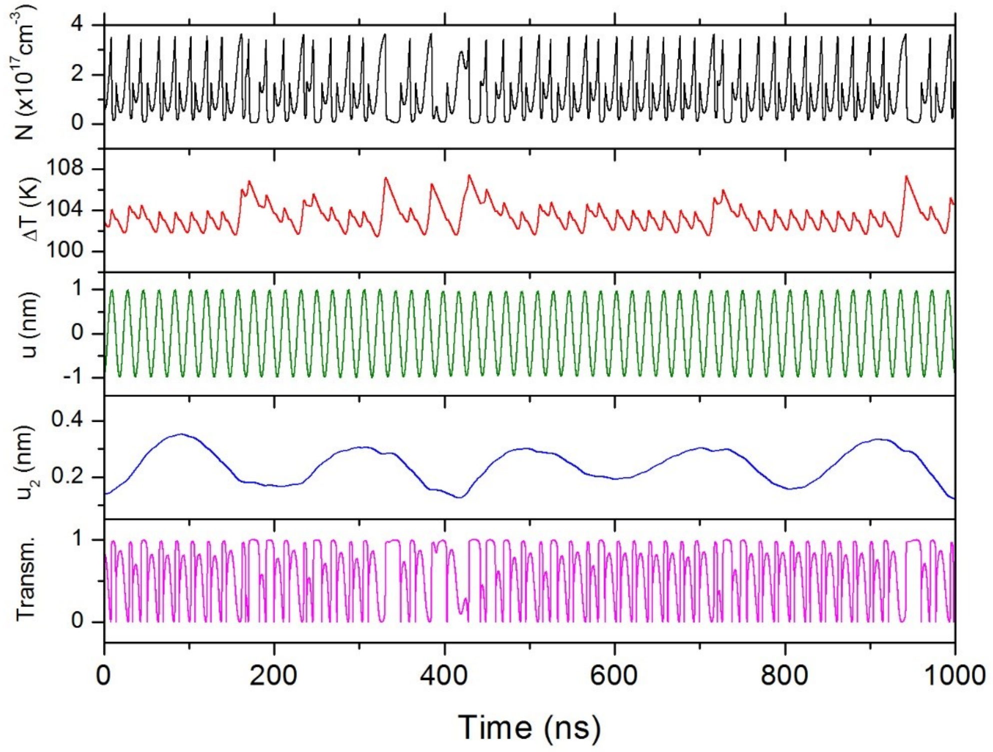

Supplementary Figure 11. Simulated temporal series of *N, ΔT, u,* u_2 and Signal Transmission in the chaotic regime considering two mechanical oscillators. From top to bottom: *N (*black), *ΔT* (red)*, u* (green), u_2 (blue) and normalised transmission (magenta). The curves are extracted in the chaotic regime (λ_l=1532.7 nm). The maximum intracavity photon number in all cases is n_o=10^5. The dynamics of *N* and *ΔT* are chaotic. On the contrary, the first oscillator (Ω_m=54 MHz) is in a coherent regime. The simulated dynamics of the second oscillator (Ω_m'=5 MHz) displays an oscillation amplitude that is high enough to display an associated peak in the frequency spectrum (see Supplementary Figure 10), but its dynamics have a little influence on the dynamics of the other magnitudes at play. The overall dynamics of the transmitted signal is chaotic, as in the experimental case.

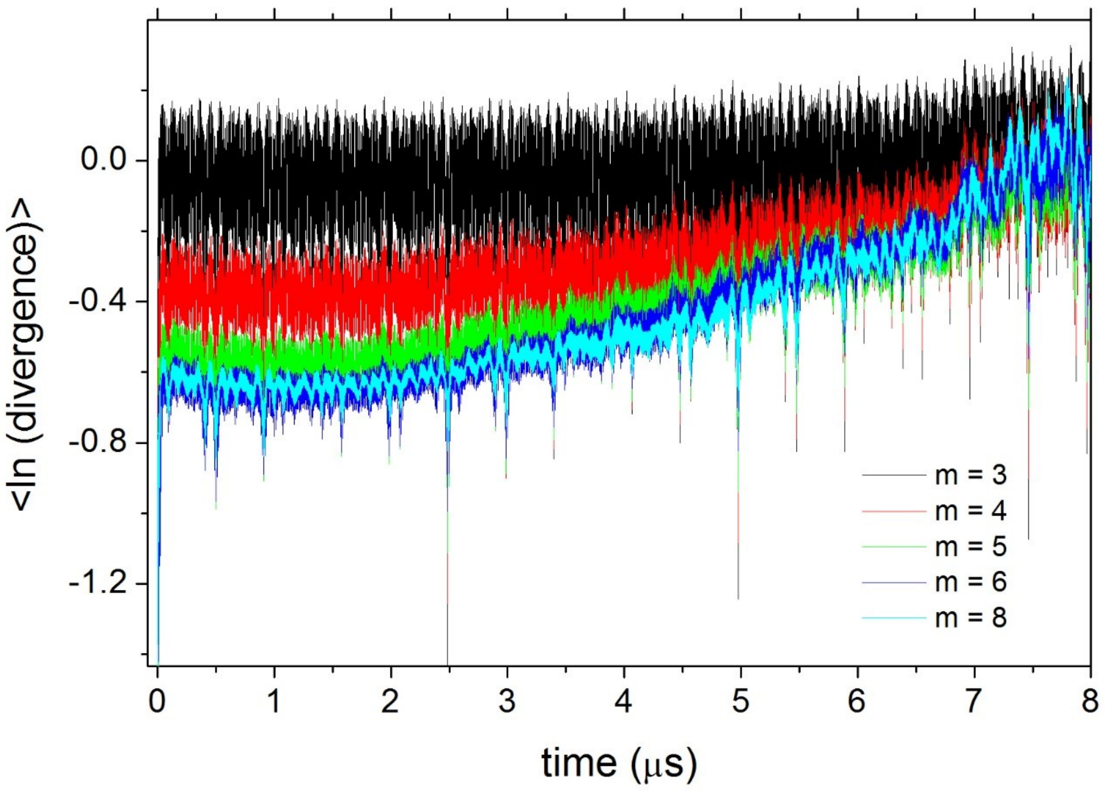

Supplementary Figure 12. Application of Rosenstein algorithm to the experimental time series in the chaotic regime. Time evolution of the <ln (divergence)> extracted from the experimental signals by applying Rosenstein algorithm for different values of the embedding dimension. The other input parameters are: delay=10 ns; mean period=20 ns. The total time register is 10 μs long, acquired with a resolution of 10 ps. Above m=6 the output traces are equivalent.

Supplementary Discussion. Comparison between our work and Ref. [J. Wu et al., arXiv:1608.05071 (2016); Reference 16 of the main text and Supplementary Reference 3]*:

Supplementary Reference 3 is one of the few experimental works claiming the observation of chaos in an OM integrated system. Hereafter we enlist several points that may allow comparing it with our works, including the mechanisms reported in the current manuscript and previous reports, that is, Supplementary Reference 7:

Comparison of geometries. Supplementary Reference 3 studies a 2D crystal by exploiting the OM interaction between a slot optical mode and a single breathing mechanical mode. On the other hand, we report on a 1D nanobeam in which several mechanical modes are at stake, enriching some of the dynamical solutions. Indeed, the chaotic states reported in Figs. 4 and 5 involve the activation of an in-plane and an out-of-plane mechanical modes, thus making the chaotic state intrinsically different from that claimed in Supplementary Reference 3. Two in-plane flexural modes are also involved in the laser power bi-stability reported on Fig. 3 of the main text.

Bistability and hystheresis. One of the main points of our manuscript is the observation of bistability and hysteresis between bidimensional and four-dimensional limit cycles, between different coherent mechanical states and between four-dimensional limit cycles and chaos. Both the laser wavelength and its power have been varied in different senses to unveil those features. We have also successfully reproduced those features by solving a numerical model of non-linear differential equations. Supplementary Reference 3 does not address bi-stability of any kind.

Comparison of basic dynamics: Pure self-pulsing and phonon lasing. The basic underlying mechanism leading to the dynamics observed in Supplementary Reference 3 is Thermo-Optic/Free-Carrier-Dispersion (TO/FCD) self-pulsing (SP) driven by two-photon absorption. However, as we acknowledge in our manuscript, this dynamics is not novel. Pure SP has been reported in several exclusively-photonic systems during the last decade (see pages 4-6 of Supplementary Reference 4 for a recent review on the topic). To the best of our knowledge, it was first observed a decade ago by Johnson et al. 5. In Supplementary Reference 1 we experimentally demonstrate that SP can couple to a mechanical mode through optical forces in an OM system. As a consequence of that coupling, we also demonstrate phonon lasing. Those experimental observations were reproduced with a numerical model, reported for the first time in our Supplementary Reference 1 in 2015, which is essentially equivalent to that of Supplementary Reference 3 (Eqs. 1-4). Reference 3 omits any reference to SP, including our work from Supplementary Reference 1.

The maximum frequency reached by the pure SP regime is similar in both systems (several tens of megahertz) as we understand it is mainly limited by heat dissipation to the surrounding atmosphere.

Comparison of the interpretation of the different dynamic regimes. By increasing the laser wavelength, the system of Supplementary Reference 10 displays unstable pulses (USP, the authors do not provide further insights), which are not present in our system. Then the system of Supplementary Reference 10 passes through several flat frequency regions at integer fractions of the mechanical modes. The interpretation of the dynamics within those regions is missing in Supplementary Reference 3. In 2015, we reported similar, though much wider, flat frequency regions in Supplementary Reference 1 at comparable laser powers together with the

result of a numerical modelling that reproduces the experimental findings and the following interpretation: "*When the mechanics/self-pulsing resonant condition is achieved, "flat regions" appear, indicating the coherent vibration of the OM photonic crystal. Since all the dynamics is coupled together, the OM oscillations provide an active feedback that stabilizes the SP. In those specific conditions, the two oscillators are frequency-entrained (FE) in a way that the SP adapts its oscillating frequency to be a simple fraction of the mechanical eigenfrequency. Similarly to the case of synchronized oscillators, the lowest M values have the largest FE zones…."*.

Comparison of OM interaction strengths. The only mechanical mode at play in Supplementary Reference 3 happens at almost twice the maximum SP frequency (that is at 112 MHz) and its OM coupling rate is stated to be $g_{o,\mathrm{OM}}/2\pi$ = 110 kHz. In our case, the main mechanical mode that is at the heart of the complex dynamics discussed along the manuscript, is an in-plane flexural mode happening at 54 MHz, which falls within the frequency range covered by the pure SP dynamics, and displaying an OM coupling rate of $g_{o,OM}/2\pi$ = 300 kHz. This two combined values enable a much stronger coupling between the SP and the mechanical harmonic oscillator. Indeed, in our work it is possible to use the first harmonic of the optical force to drive the mechanical mode. The stronger coupling appears evident when comparing the much larger frequency entrained regions (frequency plateaus) present in our case (4-6 nm in the best case) with those reported in Ref 3 (less than 1 nm in the best case).

Qualitative comparison of the chaotic dynamics and route towards chaos. Supplementary Reference 3 claims the observation of chaos at specific laser-cavity detunings. In our opinion, the authors of Supplementary Reference 3 misinterpret the transition among all the previous states as a route towards chaos. A typical route to chaos is studied, for instance, in Supplementary Reference 6, where the dynamical system starts from a limit cycle and becomes chaotic through subsequent period doubling bifurcations as an external parameter is varied. Along the route, the effective dimension of the system must be always higher than two, as stated by the Poicaré-Bendixson theorem. Contrary to what is stated by the authors of Supplementary Reference 3, a SOM state (isolated SP) cannot participate in this route because of being a solution of an effective two-dimensional system. Indeed, in our opinion, the route to chaos in the case of Supplementary Reference 3 should have been studied just in the transition between the $f_{\mathrm{OMO}}/2$ state and the chaos state. Therefore, our opinion is that Figures 3 and 4e of Supplementary Reference 3 do not report a route to chaos.

In our case, we clearly state that the transition to chaos occurs only at high enough powers, is abrupt to the best of the resolution of our tuneable laser (1 pm) and starts from the M=1 coherent state. We also observe that the transition between the M =2 and M =1 at high power follows a route along which the attractor undergoes subsequent period-doubling bifurcations (see lower part of Fig. 4a, Fig. 4b and Fig. 4c).

Comparison of intracavity optical energy thresholds to disclose chaotic dynamics. An intracavity stored optical energy (calculated at perfect laser/cavity resonance) of about 50 fJ is required for observing chaos, which is slightly lower than in Supplementary Reference 10 (60 fJ).

Quantitative comparison of the analysis of the chaotic dynamics. Authors of Supplementary Reference 3 use the Grassberger-Procaccia (GPA) method, which is known to have been the most popular method used to quantify chaos in the 80s. However, as stated in a later reference (Supplementary Reference 2), it is sensitive to variations in its parameters, for instance, number of data points, embedding dimension, reconstruction delay, and it is usually unreliable except for long, noise-free time series. Hence, the outcomes of the GPA algorithm are questionable.

The authors of Supplementary Reference 3 do not give details of the time registers in the main text, that is, number of points and total temporal length evaluated. Further details would be provided in the missing Supplementary Information document, which prevents us to do a complete analysis. Moreover, the largest Lyapunov exponent (LLE) provided by the authors of Supplementary Reference 3 is 3 times greater than the typical frequency of the system, which means that the horizon of predictability is much smaller than the typical period of the system. Just by inspection of the temporal series of Figure 2a of Figure 3d2 it is possible to realize that this cannot be the case of Supplementary Reference 3, that is, the signal should look almost stochastic and it is not.

In our case, we apply the Rosenstein algorithm, which is widely acknowledge to be accurate because it takes advantage of all the available data and works well with small data sets. In addition, it is robust where GPA is not, that is, to changes in the embedding dimension, size of data set, reconstruction delay, and noise level.

By the time of submitting the current manuscript, the Supplementary Information file of Supplementary Reference (3) has not been made public.

Supplementary References

[1] Navarro-Urrios, D. *et al.* A self-stabilized coherent phonon source driven by optical forces. *Sci. Rep.* **5**, 15733 (2015).

[2] Rosenstein, M. T., Collins, J. J. & De Luca, C. J. A practical method for calculating largest Lyapunov exponents from small data sets. *Physica D* **65**, 117{134 (1993)..

[3] Wu, J. *et al.* Dynamical chaos in chip-scale optomechanical oscillators. Preprint at https://arxiv.org/abs/1608.05071 (2016).

[4] Navarro-Urrios, D. *et al.* Self-sustained coherent phonon generation in optomechanical cavities. *J. Opt.* **18**, 094006 (2016).

[5] Johnson, T. J., Borselli, M. & Painter, O. Self-induced optical modulation of the transmission through a high-Q silicon microdisk resonator. *Opt. Express* **14**, 817{831 (2006).

[6] Bakemeier, L., Alvermann, A. & Fehske, H. Route to Chaos in Optomechanics. *Phys. Rev. Lett.* **114**, 013601 (2015).
